# The effects of vitamins C and B12 on human nasal ciliary beat frequency

**DOI:** 10.1186/1472-6882-13-110

**Published:** 2013-05-20

**Authors:** Jian Jiao, Na Meng, Hong Wang, Luo Zhang

**Affiliations:** 1 Department of Otolaryngology, Head and Neck Surgery, Beijing TongRen Hospital, Capital Medical University, Beijing, China; 2 Key Laboratory of Otolaryngology, Head and Neck Surgery (Ministry of Education), Beijing Institute of Otolaryngology, Beijing, China

**Keywords:** Ciliary beat frequency, Vitamin C, Vitamin B12, Nasal epithelial cell

## Abstract

**Background:**

This study was designed to investigate the effects of the vitamins C and B12 on the regulation of human nasal ciliary beat frequency (CBF).

**Methods:**

Human nasal mucosa was removed endoscopically and nasal ciliated cell culture was established. Changes of CBF in response to different concentrations of vitamin C or vitamin B12 were quantified by using high-speed (240 frames per second) digital microscopy combined with a beat-by-beat CBF analysis.

**Results:**

At the concentrations of 0.01% and 0.10%, vitamin C induced an initial increase, followed by a gradual decrease of CBF to the baseline level, while 1.00% vitamin C induced a reversible decrease of CBF. Vitamin B12, at the concentrations of 0.01% and 0.10%, did not influence CBF during the 20-min observation period, while a 1.00% vitamin B12 treatment caused a time-dependent but reversible decrease of CBF.

**Conclusions:**

Treatment with vitamin C or vitamin B12 caused a concentration-dependent but reversible decrease of CBF in cultured human nasal epithelial cells. Therefore, it is necessary to choose a concentration that is safe, effective, and non-ciliotoxic when applying these drugs topically in the nasal cavity.

## Background

Intranasal administration as a non-invasive route for drug delivery has generated much interest within the pharmaceutical industry in recent years. The nasal mucosa has many advantages as a potential site for both topical and systemic drug delivery, which include a large surface area for delivery, rapid onset of therapeutic effect, potential for central nervous system delivery, no first-pass metabolism, and, owing to its non-invasive nature, is likely to maximize patient comfort and compliance [1].

The safety of drug administration is an important parameter that must be taken into consideration during intranasal drug delivery. The nasal mucosa is lined by pseudostratified columnar ciliated epithelium. Coordinated beating of epithelial cell cilia with a normal pattern and frequency, which induces clearance of mucus from the airway, is the driving force of respiratory mucociliary transport. Impairment of the nasal mucociliary transport system can produce serious consequences because mucociliary transport is a major defense mechanism of the respiratory tract [2]. Therefore, investigation of the influence of nasally-administered drugs on ciliary activity is of great importance to assessing drug safety.

Vitamin C (ascorbic acid) is an important antioxidant in the respiratory tract, it is also an effective anti-inflammatory and anti-allergic agent; supplementation of vitamin C has been verified as an effective therapy for the treatment of certain respiratory diseases, including allergic rhinitis [3], chronic rhinosinusitis [4], and the common cold [5]. Since intranasal delivery route is commonly used for rhinologic drugs, we consider the intranasal route as a possible route for vitamin C supplementation, either alone or as an ingredient of certain drugs or medications.

Vitamin B12 (cobalamin) is necessary for the development of red blood cells, growth, and proper functioning of the nervous system [6]. Vitamin B12 deficiency is frequently found in elderly patients, with the main causes being pernicious anemia and food-cobalamin malabsorption. Management of vitamin B12 deficiency with cobalamin injections is currently well documented, but new routes for the administration of vitamin B12, both nasal and oral, are currently being developed [7].

The aim of the present study was to assess the ciliary toxicity of a range of concentrations of vitamin C or vitamin B12 treatment on cultured human nasal epithelial cells, so as to provide some advice for drug development and clinical medication.

## Methods

### Reagents

Dulbecco’s Modified Eagle’s Medium (DMEM), Dulbecco’s Modified Eagle’s Medium/Ham’s F12 (DMEM/F12), Hank’s balanced salt solution (HBSS) and antibiotics were purchased from GIBCO BRL (Grand Island, NY, USA). Vitamin C was provided by Prof. Zinreich (Johns Hopkins Hospital, Baltimore, USA). Vitamin B12, human placental collagen, protease, and all other chemicals were obtained from Sigma-Aldrich (St. Louis, MO, USA).

### Human nasal epithelial cell cultures

This study was performed at the Department of Otolaryngology, Head and Neck Surgery, Beijing TongRen Hospital. The study protocol was reviewed and approved by the local ethics board of Beijing TongRen Hospital, Capital Medical University. Informed consent was obtained from all the participants.

Human uncinate process was endoscopically obtained from 8 adult patients who had cerebrospinal fluid rhinorrhea, nasopharyngeal angiofibroma or nasal septum deviation, without acute rhinosinusitis, chronic rhinosinusitis or allergic rhinitis.

Primary culture of human nasal epithelial cells was established using an adaptation of the methods published by Nlend *et al.*[8]. The dissected mucosa was incubated in 0.05% protease (type XIV) in DMEM overnight at 4°C. After protease treatment, epithelial cells were released by vigorous shaking and were harvested by centrifugation. The cells were plated on collagen-coated glass coverslips (human placental collagen, type IV) at a density of 5×10^5^/ml. The medium consisted of DMEM/F12 supplemented with 10 μg/ml insulin, 5 μg/ml transferrin, 0.36 μg/ml hydrocortisone, 20 ng/ml triiodothyronine, 7.5 μg/ml endochelial cell growth supplement, 100 U/ml penicillin and 100 μg/ml streptomycin. Cells were grown in an incubator at 37°C in 5% CO_2_. The medium was changed every other day.

### Measurements of ciliary beat frequency

Ciliary beat frequency was measured on the 1st, 3rd, 7th and 14th day of cell culture. Cell cultures between the 3rd to 14th days were used for CBF measurements in response to 3 different concentrations of vitamin C or vitamin B12 (0.01%, 0.10%, 1.00%), as well as HBSS. HBSS was used as a negative control to rule out the influence of the turbulence caused by changing the solution in the cell chamber.

The cultures were first allowed to equilibrate to 25°C for 10 minutes, and initially measured for three seconds to obtain a baseline CBF by rinsing with Locke Ringer’s solution (136 mM NaCl, 5.6 mM KCl, 10 mM HEPES, 14.3 mM NaHCO_3_, 1.2 mM MgCl_2_, 2.2 mM CaCl_2_ and 11.5 mM dextrose, pH 7.35). The cultures were then treated with the appropriate concentration of vitamin C, vitamin B12, or HBSS, and CBF was continuously recorded for three seconds every 1 minute for 20 minutes.

For high concentration of vitamins, namely, 1.00% vitamin C or 1.00% vitamin B12, after CBF measurements in vitamins solution for 20-min time-period, the cultures were followed by wash-out with the Locke Ringer’s solution for another 20 minutes.

Measurements of CBF were made according to a previously published method [9,10]. Briefly, ciliated epithelial cells were viewed with an Olympus IX71 inverted microscope (Tokyo, Japan) equipped with a ×40, 1.3 NA, Ph 4, oil immersion objective. Imaging of cilia movement was carried out at 25°C and was achieved by directing the light forming the phase-contrast images to a high-speed CCD camera (TM-6710CL, Pulnix America, Sunnyvale, CA, USA). The camera was coupled with a frame grabber (Meteor, Matrox Electronic Systems, Dorval, Quebec, Canada) and recording software (StreamPix 3.16.5, Norpix Inc, Montreal, Quebec, Canada). The phase-contrast images of cilia movement were recorded at 240 frames per second. Images were evaluated using an image analysis program (IPLab v3.65a, Scanalytics, Inc., Fairfax, VA, USA).

The frequency of each ciliary beat cycle was calculated from the period of each cycle of the gray intensity waveform by a beat-by-beat analysis. The data analysis approach can measure the period [frequency (hertz) = 1/period] of each ciliary beat cycle to match the high temporal resolution of the image capture rate. The CBF was measured using a 3-second waveform (~720 frames) that was generated by the variation in gray level intensity of the phase-contrast images that resulted from the repetitive motion of the cilia. A region of interest, near the ciliary tip when the cilia were lying in the rest phase position, was selected and the average gray value was digitally extracted from the image data set and plotted with respect to time (i.e. frame number). The frequency of each ciliary beat cycle was determined from the period of each cycle of the gray intensity waveform.

### Statistics

The data were expressed as means ± SEM. Statistical significance was assessed using a Repeated Measures ANOVA followed by Bonferroni’s multiple comparison test. A value of *p* < 0.05 was considered statistically significant.

## Results

### Ciliary beat frequency on different culture day

On the 1st, 3rd, 7th, and 14th day of cell culture, CBFs of cultured cells were 7.32±1.55 Hz, 10.75±1.53 Hz, 10.73±2.15 Hz and 9.92±1.97 Hz, respectively. The CBF on the 1st day is significantly lower than the other days; in addition, the CBFs from the 3rd to 14th day are most close to the CBF of *in vivo* nasal epithelial cells, which is from 9 to 15 Hz. Therefore, we choose cell cultures from the 3rd to 14th day for CBF measurements in the following experiments.

### Changes of CBF in response to vitamin C

**Figure 1:**
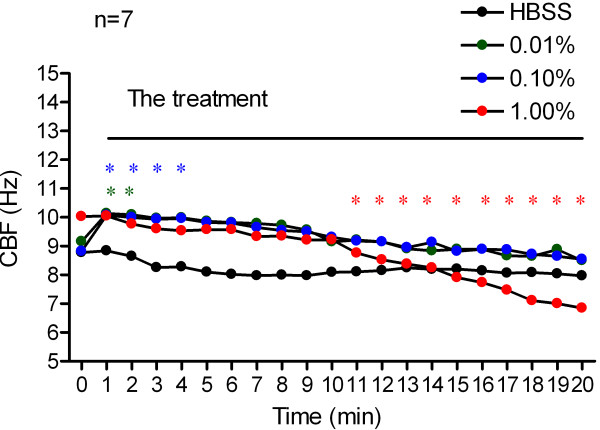
**Changes of CBF in response to vitamin C.** Ciliary beat frequency of cultured human nasal epithelial cells, measured before and every 1 min after treatment with 0.01% (*n* = 7, green dotted line), 0.10% (*n* = 7, blue dotted line) and 1.00% (*n* = 7, red dotted line) vitamin C, or HBSS (n = 7, black dotted line) over 20 minutes. *P < 0.05, vs. 0 min of each group.

The baseline CBF (CBF before treatment) for each of the three vitamin C groups and the HBSS group was 9.15 ± 1.07 Hz, 8.82 ± 1.29 Hz, 10.03 ± 1.67 Hz and 8.78 ± 1.56 Hz, respectively. There was no significant difference in baseline CBF within the groups (Figure 1).

Following treatment with 0.01% vitamin C, human nasal CBF increased over the first 2 min. The highest CBF, which was 10.13 ± 1.56 Hz, was observed at the first measurement point (1 min). CBF then gradually decreased to the baseline level. No distinguishable change in CBF relative to the baseline CBF was identified over the subsequent 18 min (Figure 1).

Similarly, 0.10% vitamin C increased CBF over the first 4 min. The highest CBF, 10.07 ± 1.77 Hz, was again observed at 1 min after treatment. CBF then gradually decreased to the baseline level. No distinguishable change in CBF relative to the baseline CBF was identified over the subsequent 16 min (Figure 1).

**Figure 2:**
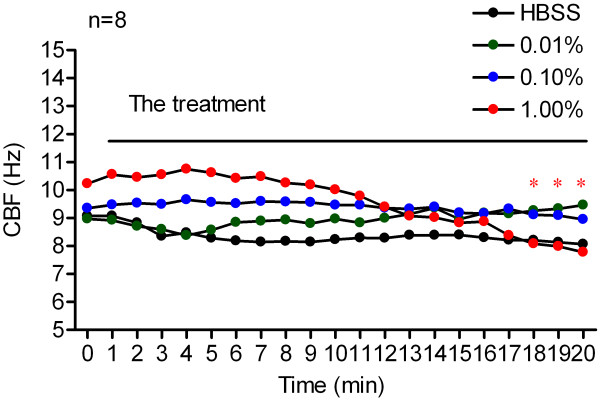
**Changes of CBF in response to vitamins wash-out.** Ciliary beat frequency of cultured human nasal epithelial cells, measured before and after treatment with 1.00% vitamin C (*n* = 5), or 1.00% vitamin B12 (*n* = 5) over 20 minutes, followed by another 20 minutes wash-out with Locke Ringer’s solution. *P < 0.05, vs. 20-min time-point of vitamin C treatment, #*P* < 0.05, vs. 20-min time-point of vitamin B12 treatment.

Ciliary beat frequency decreased significantly in response to the addition of 1.00% vitamin C. Initially, the CBF remained constant for approximately 10 min, and then continued to decrease over the next 10 min. At 20-min time-point after drug treatment, the CBF decreased to 6.84 ± 1.09 Hz (Figure 1). However, after wash-out with Locke Ringer’s solution, the CBF recovered rapidly in a time-dependent manner; CBF on 5-min time point during wash-out period recovered to 108.16 ± 3.32% of baseline CBF (Figure 2).

On treatment with HBSS, no discernible change in CBF relative to the baseline CBF was identified during the 20 minute measurement period (Figure 1).

### Changes of CBF in response to vitamin B12

**Figure 3:**
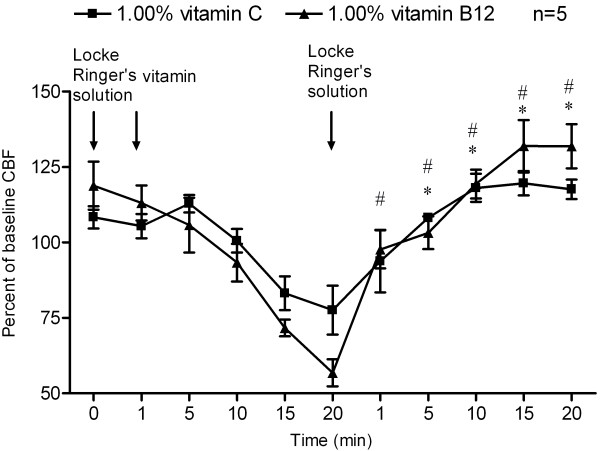
**Changes of CBF in response to vitamin B12.** Ciliary beat frequency of cultured human nasal epithelial cells, measured before and every 1 min after treatment with 0.01% (*n* = 8, green dotted line), 0.10% (n = 8, blue dotted line) and 1.00% (n = 8, red dotted line) vitamin B12, or HBSS (n = 8, black dotted line) over 20 minutes. *P < 0.05, vs. 0 min of each group.

Ciliary beat frequency changes in response to vitamin B12 are shown in Figures 3 and 2. Baseline CBF for the 0.01%, 0.10%, 1.00% vitamin B12 and HBSS groups was 8.96 ± 1.02 Hz, 9.35 ± 2.55 Hz, 10.23 ± 2.30 Hz and 9.07 ± 1.67 Hz respectively. No significant difference was found in baseline CBF within the groups (Figure 3).

Treatment with 0.01% and 0.10% vitamin B12 induced no significant change in CBF during the entire 20-minute measurement period. The CBF ranged from 8.96 ± 1.02 Hz and 9.35 ± 2.55 Hz (before treatment) to 9.46 ± 0.26 Hz and 8.95 ± 0.72 Hz (20 min after treatment) respectively (Figure 3).

At a concentration of 1.00%, vitamin B12 induced a gradual decrease in CBF and statistical significance was identified at the last three 1-minute time points. At 20 min after 1.00% vitamin B12 treatment, CBF had decreased from 10.23 ± 2.30 Hz (baseline CBF) to 7.78 ± 2.34 Hz (Figure 3).

At concentration of 1.00%, vitamin B12-induced CBF depression was also reversible, since the CBF was restored to the baseline level even after 1 minute of rinsing with Locke Ringer’s solution, CBF at 1-min wash-out period reached to 97.68 ± 14.08% of baseline CBF (Figure 2).

Following treatment with HBSS, no discernible change in CBF relative to baseline CBF was identified during the 20 minute measurement period (Figure 3).

## Discussion

Vitamin C is a well-known water-soluble and potent antioxidant, which exerts its antioxidant effect through scavenging reactive oxygen species by very rapid aqueous phase electron transfer, and preventing initiation of lipid peroxidation [11]. Vitamin C also has anti-inflammatory and anti-allergic effects; it might modulate the immune system by affecting the functions of phagocytes, proliferation of T lymphocytes, production of interferon, and gene expression of monocyte adhesion molecules [12]. In addition, vitamin C has been reported to be a biological regulator of the cystic fibrosis transmembrane conductance regulator (CFTR), which is a cAMP-dependent chloride channel that regulates respiratory tract epithelial surface fluid secretion, as well as controlling the hydration of mucosal surfaces and promoting effective mucociliary clearance. Topical administration of vitamin C to freshly-excised sinus and nasal mucosa was found to enhance chloride secretion, suggesting a potential for use as a therapeutic agent for the improvement of mucociliary clearance [13,14].

Given that oxidative damage, inflammation, immunological disturbance, and impairment of mucociliary clearance are common pathogenic factors associated with the pathogenesis of various rhinological diseases, such as chronic rhinosinusitis and allergic rhinitis, supplementation with vitamin C could have great potential as a novel therapy, either alone or in combination with traditional medications for the treatment of these diseases. In fact, some basic and clinical research has reported the effectiveness of vitamin C supplementation for the treatment of some respiratory diseases [3,4,15]. However, at present, most vitamin C delivery is by oral supplementation. Since most traditional medications for nasal disease, such as corticosteroid, antihistamines and vasoconstrictors, can be applied intranasally, we consider the intranasal delivery route as a possible route for vitamin C administration.

Vitamin B12 deficiency is common in the elderly (> 65 years of age), but is often unrecognized because of its subtle clinical manifestations, although they can be potentially serious, particularly from a neuropsychiatric and hematological perspective [16]. The main causes of vitamin B12 deficiency include pernicious anemia and food-cobalamin malabsorption, as well as poor diet, age-related gastric mucosal changes, and polypharmacy. Management of vitamin B12 deficiency with cobalamin injections is currently well-codified, but new routes of cobalamin administration (oral and nasal) are being studied, and several studies have confirmed the effectiveness of oral or nasal vitamin B12 supplementation serving as an alternative for intramuscular injection [17,18]. Compared with oral therapy, intranasal vitamin B12 supplementation could improve systemic absorption and ease of administration, avoid drug complexation with intrinsic factor in the process of oral absorption [18], and have a minimal potential for adverse systemic effects. However, although the intranasal route for vitamin B12 delivery has increasingly been used in clinical practice, the drug safety of topical form in vitamin B12 has been ignored.

Based on the common view that an effect on mucociliary clearance is one of the major areas of toxicity for nasal drug administration, the present study was designed to assess the ciliary toxicity of these two vitamins for intranasal application. CBF is a basic parameter for evaluation of ciliary activity, therefore, measurements of CBF changes in response to exogenous drugs on *in vitro* nasal epithelial cell cultures are commonly used to assess drug safety. However, the *in vitro* nasal epithelial cell culture do not represent *in vivo* nasal mucosa, it is reported that CBF of *in vitro* cultured cells may change over the culture time, moreover, the CBF between *in vivo* human nasal epithelial cells and cultured human nasal epithelial cells is different over the culture time [19]. Therefore, we first measured the CBF of cultured cells on different culture-time. Our results showed that CBF from the 3rd to 14th day is most close to the CBF of *in vivo* nasal epithelial cells, so we choose cell cultures from 3rd to 14th days for measurements of CBF in response to exogenous drugs.

We found that within concentrations from 0.01% to 0.10%, vitamin C had a stimulatory effect on human nasal CBF. However, at a higher concentration (1.00%), vitamin C produced an inhibitory but reversible effect on CBF. The effects of vitamin B12 on CBF were similar with that of vitamin C. At lower concentrations (from 0.01% to 0.10%), vitamin B12 has no significant effect on human nasal CBF. However, a higher concentration of vitamin B12, 1.00%, induced a time-dependent but also reversible decrease of human nasal CBF. These results raise the possibility of inducing loss of ciliary activity with higher concentrations of vitamins when applied intranasally, indicating the necessity of choosing a safe, non-ciliotoxic concentration when applying the vitamins topically in the nasal cavity.

The mechanisms of changes in CBF in response to vitamins treatment are still unclear. Airway CBF is mainly regulated by a variety of intracellular second messenger signaling mechanisms including cyclic adenosine monophosphate (cAMP), cyclic guanosine monophosphate (cGMP), and calcium [20]. cAMP activates an axonemal protein kinase A (PKA) to phosphorylation a dynein light chain to increase CBF; cGMP mediates a phosphorylation or dephosphorylation event to increase CBF via protein kinase G (PKG) and possibly PKA; calcium seems to increase CBF via an initial direct action on the axoneme and subsequently via cross talk to the cAMP or cGMP pathways. A significant amount of work is required to understand fully the mechanisms of regulation of CBF by these vitamins.

There are several other limitations of the current study. First, due to the short life-span of *in vitro* cultured epithelial cells, the effects of two vitamins on CBF were observed for only short period of 20 minutes; however, the use of vitamin C or B12 is not the single use but long term use in clinical medication, in addition, the present study showed a reversible decrease of CBF caused by single use of vitamins at high concentration, further indicating a possible long term ciliary toxicity in clinical use. Therefore, in the following studies, we are going to further assess the long term drug safety *in vivo* on laboratory animal models. Second, our experiments were performed on relatively normal nasal mucosa of patients, but not patients with acute or chronic rhinosinusitis. Since mucociliary transport and CBF make a difference in normal and inflammatory conditions, additional experiments for these inflammatory patients will provide more convincing results. In fact, we are in the process of performing further study on these patients so as to provide more detailed information for drug safety.

## Conclusions

In summary, to the best of our knowledge, this study is the first to assess the influence of vitamin C and vitamin B12 on CBF in primary cultured human nasal epithelial cells. We have found that vitamin C, within a range of concentrations from 0.01% to 0.10%, has a stimulatory effect on human nasal CBF, while at a higher concentration (1.00%), it produces an inhibitory but reversible effect on CBF. Low concentrations (from 0.01% to 0.10%) of vitamin B12 have no obvious effect on CBF, while at a higher concentration (1.00%), it also induced a time-dependent but reversible decrease in human nasal CBF. These results do suggest the need for choosing a concentration that is effective, safe and non-ciliotoxic when applying these agents topically in the nasal cavity.

## Abbreviations

CBF: Ciliary beat frequency; DMEM: Dulbecco’s Modified Eagle’s Medium; DMEM/F12: Dulbecco’s Modified Eagle’s Medium/Ham’s F12; HBSS: Hank’s balanced salt solution; CFTR: Cystic fibrosis transmembrane conductance regulator; cAMP: Cyclic adenosine monophosphate; cGMP: Cyclic guanosine monophosphate; PKA: Protein kinase A; PKG: Protein kinase G.

## Competing interests

The authors declare that they have no competing interests.

## Authors’ contribution

JJ participated in the design of the study, carried out the ciliary beat frequency analysis, performed the statistical analysis and drafted the manuscript. NM and HW carried out the human nasal epithelial cell cultures and performed the ciliary beat frequency measurements. LZ participated in its design and coordination, conceived of the study and helped to draft the manuscript. All authors read and approved the final manuscript.

## Pre-publication history

The pre-publication history for this paper can be accessed here:

http://www.biomedcentral.com/1472-6882/13/110/prepub
